# Prevalence of herbal and traditional medicine in Ethiopia: a systematic review and meta-analysis of 20-year studies

**DOI:** 10.1186/s13643-023-02398-9

**Published:** 2023-12-13

**Authors:** Nigatu Tuasha, Sintayehu Fekadu, Serawit Deyno

**Affiliations:** 1 Department of Biology, Hawassa College of Teacher Education, Sidama National Regional State, P. O. Box 115, Hawassa, Ethiopia; 2 School of Laboratory Sciences, College of Medicine and Health Sciences, Hawassa University, P. O. Box 1560, Hawassa, Ethiopia; 3 School of Pharmacy, College of Medicine and Health Sciences, Hawassa University, P. O. Box 1560, Hawassa, Ethiopia

**Keywords:** Complementary and alternative medicine, Herbalism, Medicinal plants, Traditional healing, Use rate

## Abstract

**Background:**

The widely accepted prevalence of traditional medicine (TM) in Ethiopia was about 80 %, of which 95 % were sourced from plants. The purpose of this study was to update knowledge of the prevalence of herbal medicine or TM in Ethiopia and describe the characteristics of the population mostly relying on herbal medicine or TM to inform health policy-makers.

**Methods:**

PubMed, Google Scholar, Hinari, Scopus, and the Directory of Open Access Journals (DOAJ) were searched. The methodological quality of each included study was assessed using the quality assessment checklist for prevalence studies. Meta-analysis was conducted using STATA version 17, and the heterogeneity between studies was assessed using *I*^2^ test statistics based on the random effect model. Forest and funnel plots were used to present the data. Subgroup analysis was done by the study population, region, and setting.

**Results:**

Thirty-six studies with a total of 16,288 participants met the inclusion criteria. Meta-analysis of the study revealed that the prevalence of herbal medicine use in Ethiopia is 46 % (95 % CI, 37–54 %), with significant heterogeneity among the studies (*I*^2^ = 99.19 %). Egger’s test for publication bias of herbal medicine use revealed significant results (Egger, *P* = 0.002) which indicates possible missing of small sample size studies. The prevalence of TM use in Ethiopia is 65 % (95 % CI, 52–77 %) with significant heterogeneity among the studies (*I*^2^ = 99.18 %). Egger’s test for publication bias of TM use revealed non-significant results (Egger, *P* = 0.275). The subgroup analysis by the study setting and the region revealed variability amongst the studies. Community-based studies and Oromia National Regional State showed higher prevalence. By population type, a higher prevalence of TM use was observed amongst children and lowest amongst malaria suspects.

**Conclusions:**

The current study revealed that TM/herbal medicine utilization remained an important source of primary healthcare in Ethiopia. In comparison to the commonly reported prevalence of TM/herbal medicine, there is a considerable decline in TM/herbal medicine prevalence. High TM/herbal medicine use tendency during pregnancy necessitates safety studies to optimize the utilization.

## Background

The World Health Organization (WHO) defines traditional medicine (TM) as “the sum total of the knowledge, skill, and practices based on the theories, beliefs, and experiences indigenous to different cultures, whether explicable or not, used in the maintenance of health as well as in the prevention, diagnosis, improvement or treatment of physical and mental illness” [1]. For a number of reasons, TM is highly valued and widely used around the world. The TM/herbal medicines with strong scientific evidence on their safety profiles, sufficient efficacy, and quality contribute to the goal of ensuring that all people have access to standard health care. This made TM/herbal medicines highly valued assets [1, 2].

Herbal medicine according to WHO is a practice which includes herbs, herbal materials, herbal preparations, and finished herbal products, that contain active ingredients parts of plants, or other plant materials, or combinations [3]. It encompasses the combination of practices of indigenous systems of medicine and several therapeutic experiences of many previous generations [4]. Main plant parts used in herbal medicine include leaves, stems, flowers, roots, and seeds [5]. Herbal medicines are asserted to cure diabetes, jaundice, hypertension, tuberculosis, mental disorders, Acquired Immunodeficiency Syndrome (AIDS), cancer, skin diseases, and many other infectious diseases [4].

The use of medicinal plants as a fundamental component of the African traditional healthcare system is the oldest with a long track record and is widely acknowledged among all therapeutic systems. In many parts of rural Africa, traditional healers prescribing medicinal plants are the most easily accessible and affordable health resource available to the local community and at times the only treatment modality that exists [6]. The TM in Africa is holistic involving both the body and the mind and traditional healers offer information, counseling, and treatment to patients and their families in a personal manner [6]. Patients’ preference, the low ratio of medical doctors to the total population, and the lack of effective modern medical treatment for some ailments in Africa are additional factors for the wider practice of traditional medicines.

According to the WHO, 70–80 % of Africans today depend either totally or partially on TM [1]. Traditional medicine is widely practiced in Ethiopia. It is used to treat diverse forms of human diseases including cancer, hypertension, diabetes, bacterial infections, parasitic infections, and many more [7–11]. Over 80 % of the Ethiopian population also relies on TM according to a report as old as 1986 [12], and more than 95% of the preparations are made from plant origin [13]. This represents the majority of the rural population and sectors of the urban population where there is little or no access to modern health care [14].

The recent study in 2016 by the WHO’s Study on Global Ageing and Adult Health (SAGE) revealed that the widely accepted notion that 80 % of Africans and Asians rely on TM stands no more [15]. For instance, it was <3 % in Ghana and <2 % in South Africa, which is much less than what previous reports claimed [15]. The present systematic review and meta-analysis aimed to update knowledge of the prevalence of TM/herbal medicine in Ethiopia. In addition, it aims to point out the characteristics of the population mostly relying on TM/herbal medicine to inform health policy-makers to harness its potential contribution to health, wellness, and people-centered healthcare and promote safe and effective use through the regulation of products, practices, and practitioners as indicated in the WHO TM strategy 2014–2023 [1].

## Materials and methods

### Search strategy

Published and unpublished research papers (e.g., MSc/PhD thesis) reporting the prevalence of TM/herbal medicine in different settings: during pregnancy, for infants (children), for adults, in urban settings, and in rural settings even involving health professionals at the institution or community level were included. The search domains included Google Scholar, local university repositories—for unpublished research, international abstracting, and indexing databases such as SCOPUS, PubMed, Hinari, ScienceDirect, Web of Science, EBSCO, and Directory of Open Access Journals (DOAJ).

In designing the search strategy, participants, intervention, comparator, and outcome (PICO) were used. The participants of this study were the Ethiopian population. The intervention is TM including herbal medicine. Since this is the prevalence comparator, the outcome was not used in designing the search term.

The key terms/phrases used for searching were Ethiopia, plants, medicinal plants, traditional medicine, traditional knowledge, herbs, indigenous knowledge, folk medicine, ethnobotany, ethnopharmacology, ethnomedicine, medico-cultural, prevalence, proportion, and use rate. Based on the information above, the following search terms were applied in different databases. (1) Traditional medicine OR medicinal plant* OR herb* OR indigenous knowledge OR traditional knowledge OR folk medicine OR folk remedies OR home remedies OR ethnobotan* OR ethnopharmacolog* OR ethnomedicin* ethnopharmaceutic* OR medico-cultural; (2) Prevalence OR proportion OR Use rate; and (3) Ethiopia*. The search maps used in PubMed were as follows: ((traditional medicine OR medicinal plant* OR herb* OR indigenous knowledge OR traditional knowledge OR folk medicine OR folk remedies OR home remedies OR ethnobotan* OR ethnopharmacolog* OR ethnomedicin* ethnopharmaceutic* OR medico-cultural) AND (prevalence OR proportion OR use rate)) AND (Ethiopia*).

First, the titles and abstracts were screened and then suitable articles were downloaded and examined against the inclusion criteria. Published and unpublished ethnobotanical and ethnomedicinal surveys reporting the prevalence or proportion of the population using herbal medicine or TM in Ethiopia were included. Review articles, historical documents, experimental studies, data lacking information on study areas, prevalence of use, and not reporting information about traditional medicinal plants were excluded.

### Risk of bias assessment

The methodological quality of each included study was assessed using the quality assessment tool for prevalence studies developed by Leboueuf-Yde and Lauritsen and then modified by Hoy and colleagues [16, 17]. Graphs of the summary of the risk of bias were developed using RevMan 5.3 (Cochrane Informatics and Knowledge Management Department, London, UK).

### Data extraction

Data were extracted using a Microsoft Excel spreadsheet. The characteristics of extracted data in each study include first author name, year of publication, area of study (region), study setting (community or institution-based), number of study participants, response rate, characteristics of study participants (population type), age of study participants, frequency of herbal medicine use, frequency of TM use, and most frequently used herbal medicine.

### Data analysis

Statistical analyses were conducted using STATA version 17.0 (StataCorp, LP, College Station, TX). The prevalence was pooled using the MetaProp command in STATA. The heterogeneity of the studies was assessed using the *I*^2^ statistic, and significance was declared at *I*^2^ > 50 %. Because of significant heterogeneity among the studies, the random-effects model (REM) was used to estimate the pooled prevalence and 95 % CI using the DerSimonian and Laird methods. The Freeman-Turkey double arcsine transformation was used to avoid missing prevalence near or at 0 and 1 from the meta-analysis. Subgroup analysis was done by region, study setting, and population type. The presence of publication bias was tested using Egger’s test. Forest and funnel plots were constructed to display the individual studies and pooled results. The data were computed by Cochrane Collaboration’s software, RevMan 5.3.

## Results

### Characteristics of included studies

**Fig. 1:**
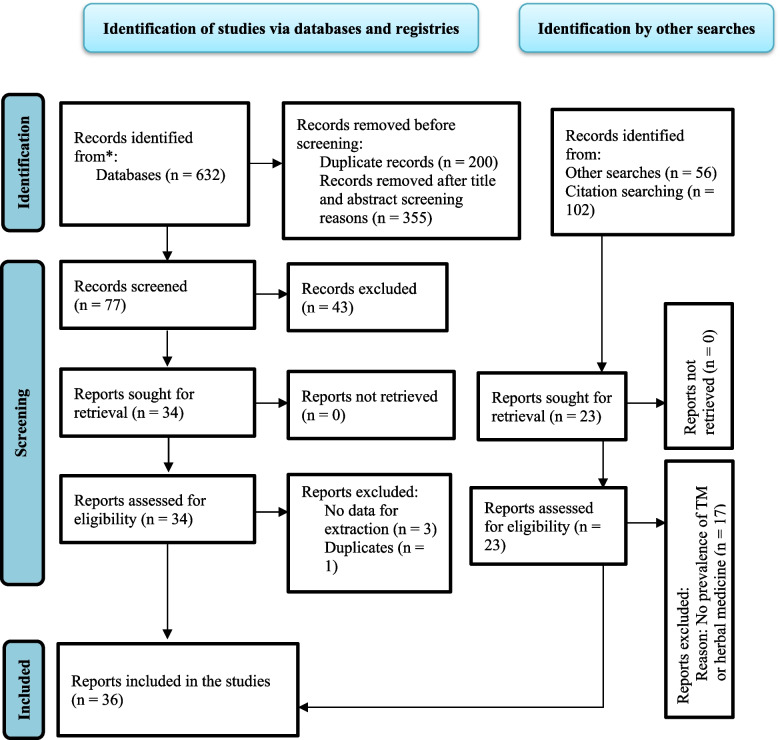
PRISMA flow diagram depicting studies identified, screened, selected, and included

Database and other relevant source searching resulted in 790 articles of which 36 met the inclusion criteria, enrolling a total of 16,288 participants. The included studies published were for 20 years from 2002 to 2022 (Fig. 1).

Of these studies, 16 were from the Amhara National Regional State; nine from Oromia National Regional State; three from Addis Ababa City Administration; three from Harari Peoples’ National Regional State; two from South Nations, Nationalities and Peoples Regional State; and one each from Southwest Ethiopia National Regional State, Benshangul-Gumuz National Regional State and in health centers from Addis Ababa and Bati, North Central Ethiopia. Twenty of the studies were conducted at institutions such as hospitals and health centers, and the remaining 16 studies were community-based studies. Twelve studies were conducted on pregnant women; nine in the general population, four in parents with children, three among HIV/AIDS patients, two among diabetic patients, two among hypertensive patients, and one each in HIV/AIDS and TB co-infected patients, cancer patients, malaria suspected patients, and among older adults. The general characteristics of included studies; regions where the studies were conducted, population type, the study setting, sample size, response rate, age groups, prevalence of herbal medicine, and TM use; and most frequently used herbal medicines were depicted in Table 1.

**Table 1:** The characteristics of the included studies

**S. №**	**Author, year of publication**	**Region/city administration**	**Population type, setting, and the study design**	**Sample size**	**Response rate (%)**	**Age ( in years)**	**Prevalence of TM use (*N*)**	**Prevalence of herbal medicine use (*N*)**	**Perceived main reasons for TM/herbal medicine use**	**Most frequently used herbal medicine**
	Abeje et al. [18], 2015	Amhara	Pregnant women, urban, health facility-based	510	100	26.5 ±6.0	40	39	Previous use experience	*Zingeber officinale* (ginger), *Allium sativum* (garlic or *Nechi shinkurit*), *Zehneria scabra* (*aregresa*), *Hageenia abyyssinica* (*kosso*), and *Cucurbita pepo L* (*Duba*)
	Addis et al. [19], 2021	Amhara	Pregnant women, urban, community-based	267	98.2	32.68± 6.47		95	Ease of availability	*Zingiber officinale* (Ginger), *Ruta chalepensis* ( *Tenaadam*), *Linum usitatissimum* ( *Telba*/linseed), *Eucalyptus globulus* ( *Nechi Bahrzaf*), and *Moringa stenopetala* (*shiferaw*)
	Ahmed et al. [20], 2020	Oromia	Pregnant women, urban, hospital-based, cross-sectional	1117	98.6	>18		301	Previous use experience, religion, and distance to a health facility	*Linum usitatissimum* ( *Telba*/linseed) and *Zingiber officinale* ( *Zingbil/*Ginger)
	Ahmed et al. [21], 2021	Oromia	Pregnant women, urban, hospital-based, cross-sectional	1117	98.6	>18		319	Lack of access to modern health facilities	*Linum usitatissimum* (flaxseed), *Ocimum*. *lamiifolium* (*damakesie*) and *Carica papaya* (*papaya*)
	Asrat et al. [22], 2020	Amhara	Parents who have children <18 years, TM use in children; community-based, cross-sectional	858	100	>18	693	393	Ease of accessibility	-
	Ayele et al. [23], 2017	Amhara	Elderly patients with chronic illnesses, institution-based cross-sectional	369	87.8	≥65	240	121	Dissatisfaction with conventional therapy	*Zingiber officinale* (Ginger) and *Allium sativum* (Garlic)
	Bantie et al. [24], 2019	Amhara	Mothers with children < 5 years with pneumonia, hospital-based, cross-sectional	173	89.4	>16		114	Residence of mothers (access)	-
	Bayisa et al. [25], 2014	Oromia	Pregnant women, hospital-based, cross-sectional	250	100	>18		250	Ease of access	*Zingiber officinale* (Ginger) and *Allium sativum* L. (Garlic)
	Emiru et al. [26], 2021	Amhara	Pregnant women, institution-based cross-sectional	282	100	>18	252	146	Accessibility and availability	*Zingiber officinale* (Ginger), *Allium sativum* (Garlic), and *Ocimum lamiifolium* (*demakese*)
	Erku and Mekuria [27], 2016	Amhara	Hypertensive patients, urban, hospital-based	423	97.39	57.32 ± 10.57	279	189	Dissatisfaction with conventional medicine	-
	Erku [28], 2016	Amhara	Cancer patients, urban, hospital-based	231	84.4	>18	154	140	belief in the advantages of CAM	-
	Feyissa et al. [29], 2022	Benishangul Gumuz	HIV/AIDS and tuberculosis co-infected patients, health facility-based, cross-sectional	412	100	37.1 ±10.4		217	Improving general wellbeing, the perception that herbal medicines are natural and safe and improving appetite	*Allium sativum* (Garlic) and *Zingiber officinale* (Ginger)
	Gedif and Hahn [30], 2002	Addis Ababa	Urban, households	600	100	>18		222	Dissatisfaction with the services of modern health institutions due to their time-consuming nature and modern medicine was too expensive	*Zingiber oficinale* (*Zingible*), *Tavarnera abyssinica* (*dingetegna*), *Lipidium sativum* (*feto*), *Ocium lamifolium* (*damakesse*), and *Ruta chalpensis* (*tena adam*)
	Gedif and Hahn [31], 2003	SNNPR	Rural, households	600	100	>18		75	Perceived efficacy, economic and geographic accessibility	*Taverniera abyssinica*, *Ocimum lamiifolium*, *Allium sativum*, *Ruta chalepensis*, *Linum usitatissimum*, *Hagenia abyssinica*, *Zingiber officinale* Rosc., and *Lepidum sativum*
	Gurmu et al. [32], 2017	Amhara	HIV/AIDS patients, urban, hospital-based, cross-sectional	300	100	>18	131	48	Religious practice and the belief and desire to improve immunity	*Nigella sativa* (*Black cumin*), *Moringa oleifera* (*Morniga*), *Allium sativum* (*Garlic*), and *Zingiber officinale* (*Ginger*)
	Haile et al. [33], 2017	Amhara	HIV/AIDS patients, urban, hospital-based, cross-sectional	396	90.9	32.5 ± 8.6		255	Dissatisfaction with conventional therapy and belief in the advantages of herbal medicines	*Zingiber officinale* (Ginger), *Allium sativum* L. (Garlic), and *Moringa stenopetala* (*shiferaw*)
	Shiferaw et al. [34], 2020	Addis Ababa	HIV/AIDS patients, urban, hospital-based, cross-sectional	318	100	43.8 ±11.4		83	To treat opportunistic infections, to reduce the antiretroviral drugs side effects and improve the wellbeing	*Allium sativum* (garlic), *Ocimum lumiifolium* ( *Damakase*), and *Linum usitatissimum* (flaxseed)
	Tizazu et al. [35], 2020	Amhara	Community-based, cross-sectional, TM use for children	374	93.90	38 ± 9.80	317	175	Accessibility	-
	Tesfaye et al. [36], 2022	Southwest Ethiopia	Pregnant women, community-based, cross-sectional	680	98	>15	247	158	Its accessibility and affordability (low/no cost)	-
	Nigussie et al. [37], 2022	Harari	Community-based, cross-sectional	818	98.2	41.05 ± 15.36	563	563	Closeness to the service and affordability	*Ocimum lamiifolium* ( *Damakase*), *Zingiber officinale* ( *Zingibil*), *Aloe megalacantha* ( *Eret*), and *Lepidium sativum* ( *Feto*)
	Nega et al. [38], 2018	Addis Ababa and Bati,	Pregnant women, health center-based, cross-sectional	624	96	18–40		360	To promote health and wellbeing	*Ocimum lamiifolium*, *Zingiber officinale, Allium sativum*, *Nigella sativa*, and *Ruta chalepensis*
	Mekuria et al. [39], 2017	Amhara	Pregnant women, urban, hospital-based, cross-sectional	410	88.8	26 ±5.0		177	Cheap, accessible, and safe	*Zingiber officinale*, *Trigonella foenum-graecum L* ( *Absh* (*fenugreek*)), *Ocimum lamiifolium* ( *damakasse*), *Allium sativum*, and *Linum usitatissimum* ( *telba* (Flax seeds))
	Mekuria et al. [40], 2018	Amhara	Type 2 diabetic patients, urban, hospital-based, cross-sectional	408	94.8	52.5 ± 12.6		240	Dissatisfaction with the modern therapy and beliefs in the merits of herbal medicines	*Allium sativum* L., *Caylusea abyssinica* (Fresen.) (*Giesilla*), *Otostegia integrifolia* Benth ( *Tinjute*), and *Hagenia abyssinica* ( *Kosso*)
	Jambo et al. [41], 2018	Harari	Pregnant woman, hospital-based, cross-sectional	247	98.8	Median age 25		142	Fewer side effects and its effectiveness	*Zingiber officinale* *Ruta chalepensis* *Allium sativum* *Ocimum lamiifolium* *Thymus vulgaris* ( *Tosign*)
	Kebede et al. [42], 2009	Addis Ababa	Pregnant woman, urban	1268	87.9	Mean age 26		28	-	*Allium sativum*, *Ocimum lamiifolium*, *Lepidium sativum*, *Cucurbita pepo, Linum usitatissimum*, *Echinops kebericho*, *Glinus lotoides*, *Ruta chalepensis*, and* Zingeber officinale*
	Kifle et al., [43] 2021	Amhara	Hypertensive patients, hospital-based, cross-sectional	475	94.7	46.54 ± 12.6	275	167	Dissatisfaction with modern medicine, belief in the advantages of CAM, and availability	*Moringa stenoptela* (*Shiferaw*) *Ocimum lamiifolium* (*Damakase*) *Calpurnea aurea* (*Digita*) *Rumex nepalensis* (*Tullet*) *Menthax piperata* (*Nana*)
	Kifle et al. [44], 2021	Amhara	Diabetic patients, institution-based, cross-sectional	419	94.3	48.7 ± 12.6		231	Dissatisfaction with allopathic medicine, traditional, or cultural acceptability, family, and belief in TM	*Moringa stenoptela* (*Shiferaw*) *Nigella sativa* (*Tikur Azmud*) *Zingiber officinale* (*Zingibil*) *Allium sativum* (*Nech Shinkurt*) *Aloe vera* (*Eret*)
	Kovalev and Wells [45], 2020	Oromia	Malaria suspected cases, community-based, cross-sectional	366	100	>15	145	116	Modern healthcare facilities are too far, unaffordable, have side effects and also there is no better expectation; TM use saves time	*Allium sativum*, *Zingiber officinale*, *Ajuga intergrifolia* (*Harmaguse*), *Allium cepa* (Onion), *Lepidium sativum* (Feto), and *Ocimum lamiifolium*
	Laelago et al. [46], 2016	SNNPR	Pregnant women, urban, health facility-based, cross-sectional	363	97	25.4 ±4.1		258	Safe, cheap, accessible, and effective	*Allium sativum*, *Zingiber officinale*, *Ruta graveolens*, and *Ocimum lamiifolium*
	Hailu et al. [47], 2020	Oromia	Parents who have children < 18 years, community-based, cross-sectional	267	100	>20	182	73	Effectiveness and satisfaction with TM; dissatisfaction, inaccessibility, and cost of modern medicine	-
	Aragaw et al. [48], 2020	Amhar	Community-based, cross-sectional	404	99.5	35.73 ± 0.59	145	144	Accessibility and affordability	-
	Belachew et al. [49], 2017	Oromia	Community-based, cross-sectional	302	100	Mean age 46	224	213	Affordability and accessibility	-
	Bussa and Gemeda [50], 2018	Harari	Community-based, urban, cross-sectional	423	100	>18	256	119	Availability and accessibility	*Allium sativum* and *Ocimum lamiifolium*
	Chali et al. [51], 2021	Oromia	Community-based, cross-sectional	271	100	>18	221	145	Affordability and religious affiliation	-
	Gari et al. [52], 2015	Oromia	Community-based, cross-sectional, urban setting	282	100	Mean age 30	265	154	It is cheap	-
	Misha et al. [53], 2014	Oromia	Community-based, cross-sectional	151	100	>15		120	Affordability, accessibility, and acceptability	-

### Quality assessment of the included studies

**Fig. 2:**
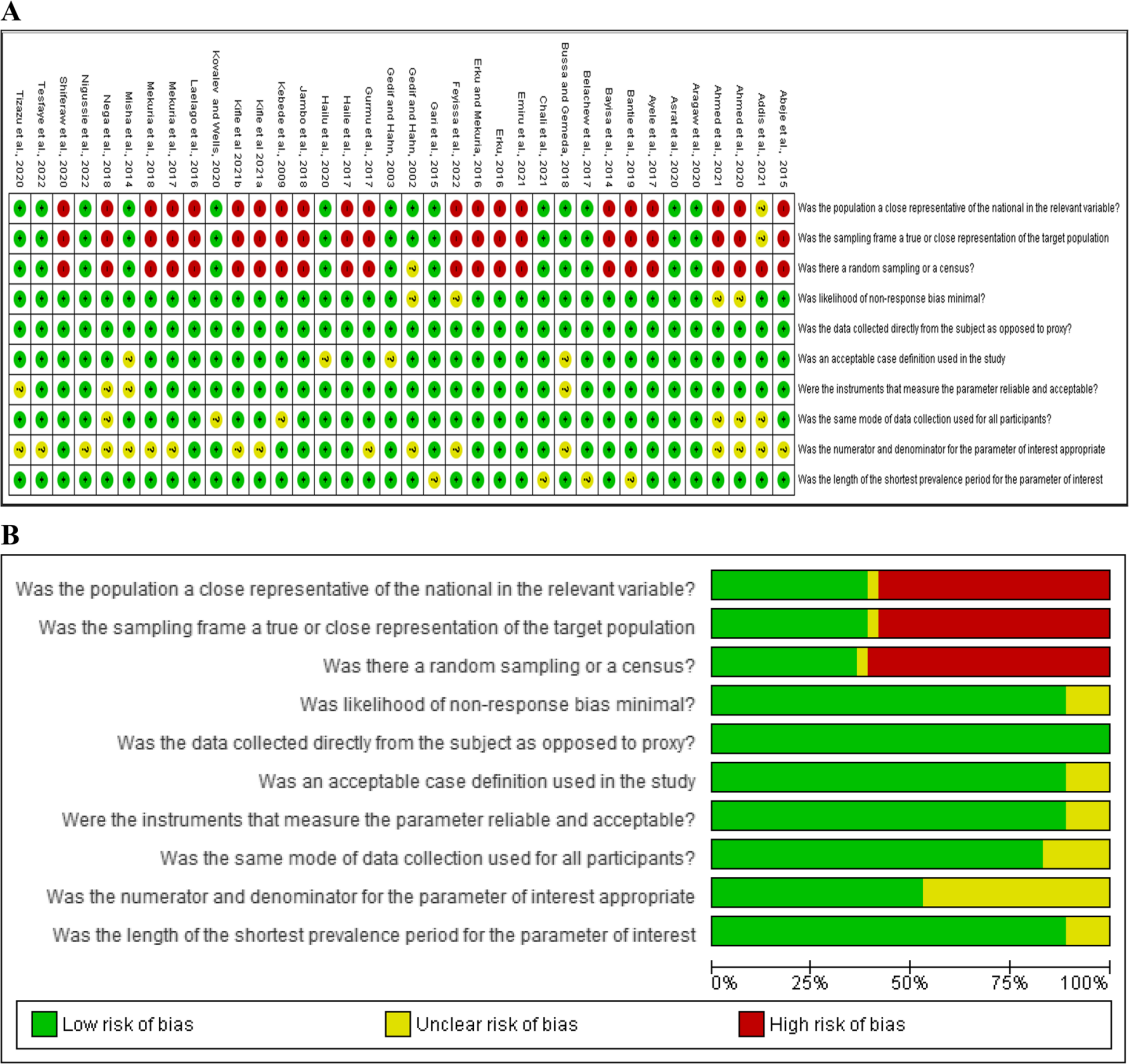
Risk of bias assessment. Risk of bias summary (**A**). Risk of bias graph (**B**)

A low risk of bias was observed in all of the included studies in terms of the source of data collection as all of them were directly collected from the participants. In terms of non-response bias, acceptable case definition, and instrument used, almost all studies have shown a low risk of bias. About half of the included studies have shown a high risk of bias in terms of representing the national population, sampling frame, and random sampling technique. The risk of bias assessment summary and the graph are presented in Fig. 2A and B.

### Prevalence of herbal medicine use in Ethiopia

**Fig. 3:**
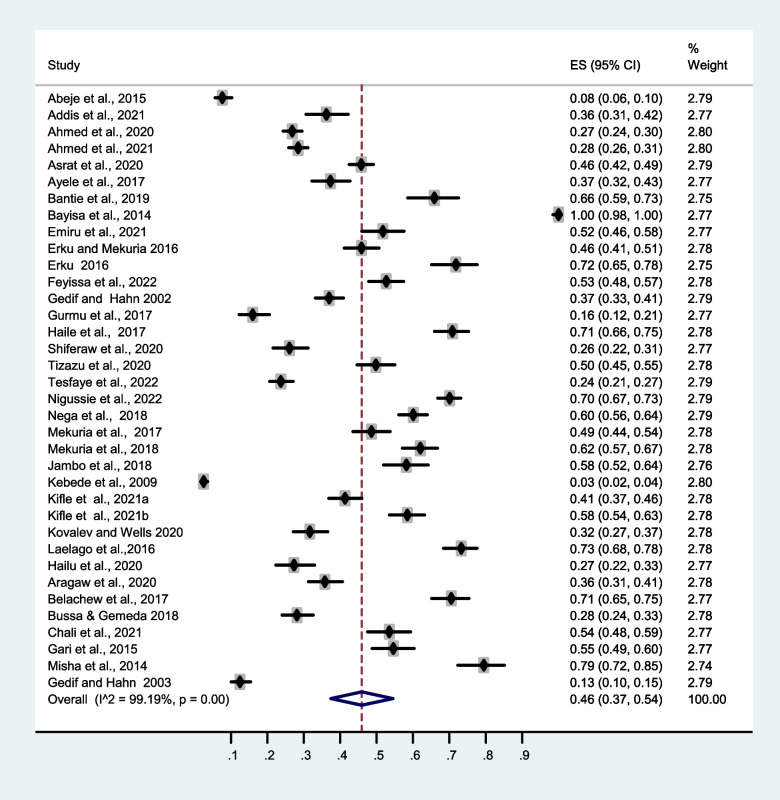
Forest plot depicting the prevalence of herbal medicine use in Ethiopia

Meta-analysis of the study revealed that the prevalence of herbal medicine use in Ethiopia is 46 % (95 % CI, 37–54 %), and there was significant heterogeneity among the studies, *I*^2^=99.19 % (Fig. 3). Egger’s test for publication bias revealed significant result (Egger, *P*= 0.002) which indicates possible missing of small sample size studies.

**Fig. 4:**
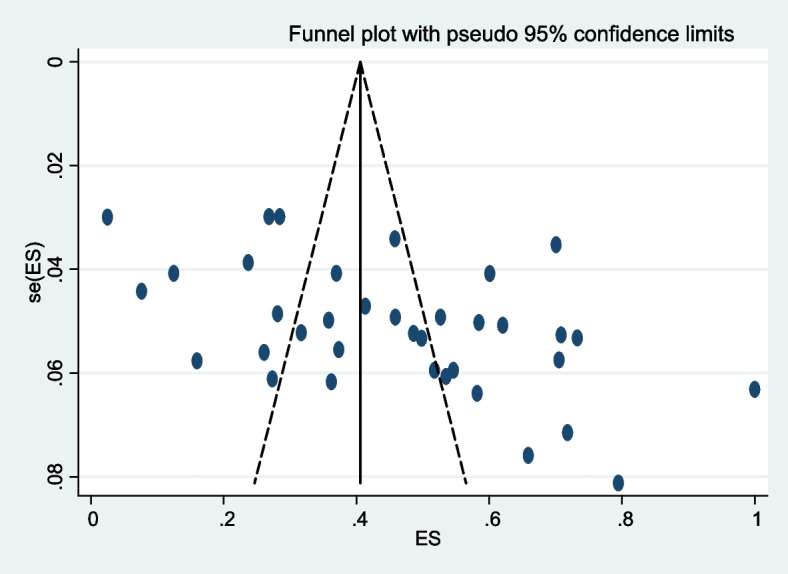
Funnel plot of prevalence of herbal medicine use distribution effect size estimation

The studies included were significantly heterogeneous as the statistical test revealed; visual inspection of the funnel also revealed the scatted distribution of the prevalence values (Fig. 4).

Subgroup analysis of the study by region revealed that there is heterogeneity among studies as indicated in Table 2. The mixed (Addis Ababa and Bati) showed a higher prevalence of herbal medicine use compared to all other regions while Addis Ababa showed the lowest prevalence. Community-based studies showed a higher prevalence of herbal medicine use compared to institutional-based studies. More studies were conducted among pregnant women, higher prevalence of use of herbal medicine was observed amongst cancer patients and lowest among malaria suspects.

**Table 2:** Results of subgroup analysis of herbal medicine use prevalence by region, study setting, and population type

**Categories**	**Subgroups**	**Prevalence (95 % CI)**	**No of studies (%)**	***I***^**2**^
Region	Mixed (Addis Ababa and Bati	60% (56%, 64%)	1 (2.78)	
	Oromia	55 % (36%, 74%)	9 (25.0)	99.32%
	Benshangul-Gumuz	53 % (48%, 57%)	1 (2.78)	-
	Harari	52 % (25%, 78%)	3 (8.33)	-
	SNNPR*	33 % (30%, 36%)	2 (5.56)	-
	Amhara	46 % (36%, 55%)	16 (44.4)	98.24%
	Southwest Ethiopia	24 % (21%, 27%)	1 (2.78)	-
	Addis Ababa	19 % (1%, 50%)	3 (8.33)	-
Setting	Community-based	45 % (35%, 56%)	16 (44.44)	99.36%
	Institutional-based	46 % (34%, 60%)	20 (55.56)	98.67%
Population type	Cancer patients	72 % (65%, 78%)	1 (2.78)	-
	Diabetic patients	60 % (57%, 64%)	2 (5.56)	.
	General populations	50 % (32%, 68%)	8 (25.0)	98.24%
	HIV/TB coinfected patients	53 % (48%, 57%)	1 (2.78)	-
	Pregnant women	43 % (26%, 61%)	12 (33.33)	99.58%
	Hypertensive patients	43 % (40%, 47%)	2 (5.56)	
	Parents with children	43 % (25%, 62%)	4 (11.11)	-
	Elderlies	37 % (32%, 43%)	1 (2.78)	-
	HIV/AIDS	37 % (8%, 71%)	3 (8.33)	
	Malaria suspects	32 % (27%, 37%)	1 (2.78)	-

SNNPR, Southern Nations, Nationalities and Peoples Region

**Fig. 5:**
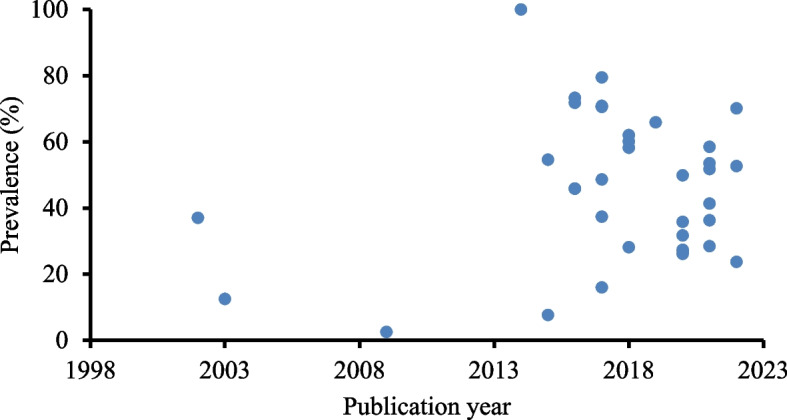
Trends in herbal medicine use in Ethiopia

The trends analysis revealed that most of the studies (91.7 %) were conducted between 2014 and 2022. The heterogeneity was visible regardless of the year of the study (Fig. 5).

### Prevalence of traditional medicine use in Ethiopia

**Fig. 6:**
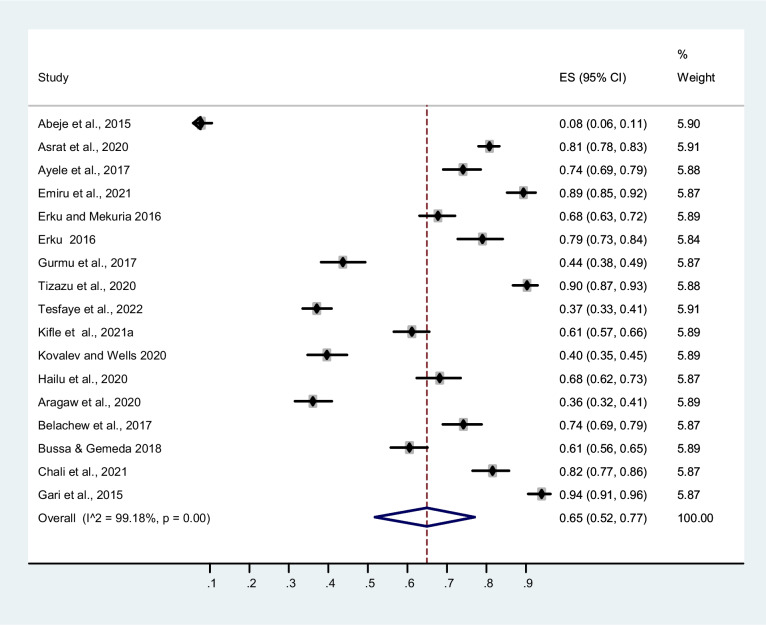
Forest plot depicting the prevalence of traditional medicine use in Ethiopia

Meta-analysis of the study revealed that the prevalence of TM use in Ethiopia is 65 % (95 % CI, 52–77 %). There was significant heterogeneity among the studies, *I*^2^=99.18% (Fig. 6). Egger’s test for publication bias revealed non-significant results (Egger, *P*=0.275).

**Fig. 7:**
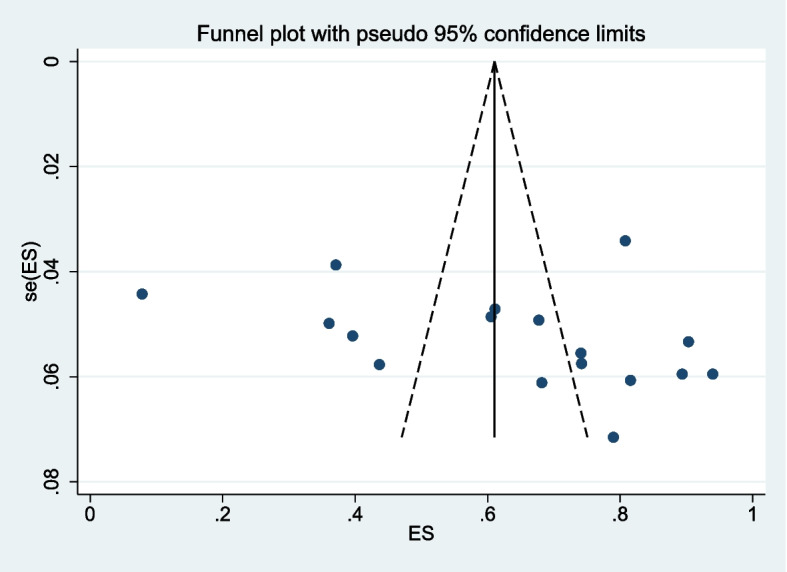
Funnel plot of prevalence of traditional medicine use distribution effect size estimation

The studies included were significantly heterogeneous as it was for the prevalence of herbal medicine use; visual inspection of the funnel also reveals a scatted distribution of the prevalence values (Fig. 7).

Subgroup analysis of TM use by region revealed that there is variability among studies as indicated in Table 3. Community-based studies showed a higher prevalence of TM use compared to institutional-based studies though non-significant. The Oromia National Regional State showed a higher prevalence of TM use compared to all other regions while Southwest Ethiopia showed the lowest use prevalence. A higher prevalence of TM was observed among children and lowest among malaria suspects.

**Table 3:** Results of subgroup analysis of traditional medicine prevalence by region, study setting, and population type

**Categories**	**Subgroups**	**Prevalence (95 % CI)**	**No of studies**	***I***^**2**^
Region	Oromia	73% (53%, 90%)	5 (29.41)	98.59%
	Amhara	64% (44%, 81%)	10 (58.82)	99.37%
	Mixed	65% (52%, 77%)	1 (5.88)	-
	Southwest Ethiopia	37% (34%, 41%)	1 (5.88)	-
Setting	Community-based	68% (53%, 81%)	10 (58.82)	99.02%
	Institutional-based	60% (36%, 83%)	7 (41.18)	99.37%
Population type	Mother/parent with children	81% (69%, 90%)	3 (17.65)	-
	Cancer patients	79% (73%, 84%)	1 (5.88)	-
	Elderly patients	74% (69%, 79%)	1 (5.88)	-
	General populations	71% (49%, 89%)	5 (29.41)	98.86%
	Hypertension	64 % (61%, 67%)	1 (5.88)	
	HIV/AIDS patients	54% (51%, 58%)	2 (11.76)	-
	Pregnant	44% (6%, 87%)	3 (17.65)	-
	Malaria suspects	40% (35%, 45%)	1 (5.88)	-

### Trends in traditional medicine use in Ethiopia

**Fig. 8:**
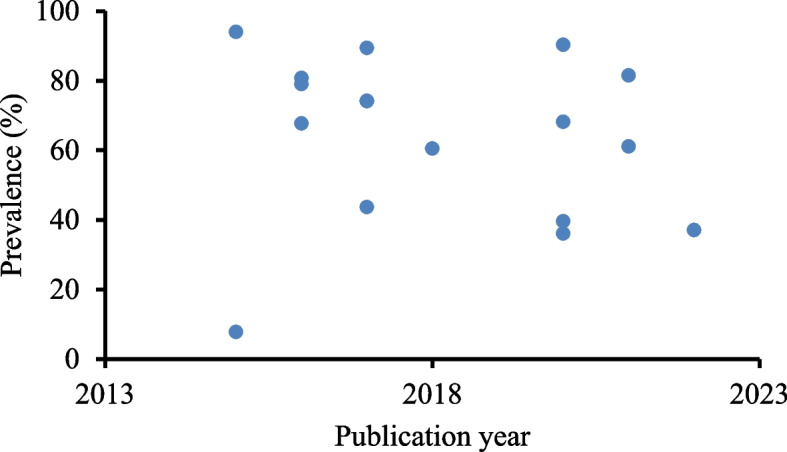
Trends in traditional medicine use in Ethiopia

Trends analysis revealed that most of the studies were conducted between 2016 and 2022. Heterogeneity is visible regardless of the year of study (Fig. 8).

## Discussion

The present finding revealed that the prevalence of 65 % (95 CI %, 52–77 %) TM and that of the herbal medicine prevalence of 46 % (95 CI %, 38–56 %) are much less than the previously established prevalence. The long-standing value that 80 % of Ethiopians rely on TM and of which 95 % is sourced from herbal medicine is far from the present truth. Traditional medicine is an integral part of healthcare as an alternative healthcare delivery system especially in low- and middle-income countries encompassing Latin America, Africa, and Asia [1, 54, 55]. In Ethiopia, according to the official population projection of the Central Statistical Agency (CSA) 2019, 79.77 % of the Ethiopian population lives in rural parts of the country [56]. The rural area is characterized by poor infrastructural settings with scarce or no modern facilities to provide primary healthcare. The TM is sometimes the only accessible and affordable alternative primary healthcare among such rural communities [1]. African TM/herbal medicine is used for various human ailments such as cancer, hypertension, HIV, and use during pregnancy follows similar trends to that of Ethiopian TM/herbalism [57–60].

Among the herbal medicines reported in the present study, there are ubiquitously used herbs as foods or dyes by the general population in daily lives. For instance, *Zingiber officinale* (Ginger), *Ruta chalepensis* (*Tena Adam*), *Allium sativa* (Garlic), *Ocimum lamiifolium* (*Damakase*), *Thymus vulgaris* (*Tosign*), *Lepidium sativum* (*Feto*),* Trigonella foenum-graecum L. *(*Absh *(*fenugreek*)**, **and* Linum usitatissimum *(*telba *(Flax seeds)) are among the reported herbal medicines and further contributed to the increase in the prevalence [61, 62]. These are common dietary supplements/spices and are also commonly used herbs for medicinal purposes.

The trend analysis of the studies revealed that the majority of the studies with proportions of herbal medicine use were conducted between 2014 and 2022, the last 8 years (Table 4). This finding further signifies that the old figures of TM/herbal medicine prevalence needed updating and hence the present finding can be referred to as the current prevalence of TM/herbal medicine in Ethiopia.

The present finding that a lower socio-economic status, unemployment, and rural residence where access to modern health facilities is scarce were associated with high TM/herbal medicine use is in agreement with other reports [63, 64]. The WHO’s study on Global Ageing and Adult Health (SAGE) also determined that the TM prevalence in six populous middle-income countries such as China, Ghana, India, Mexico, Russia, and South Africa is much lower than has previously been reported and those who do make use of TM are more likely to be socio-economically disadvantaged corroborates the present finding [15].

The most regularly cited reasons for TM/herbal medicine use in the present finding disclosed are closeness to residency, cultural acceptability, trust by the general population, ease of access, affordability, and dissatisfaction with modern medicine also supported by other reports elsewhere [15, 63–65]. In some high-income countries, the TM usage is reported to be high. For instance, Australia (48 %), Canada (70 %), France (75 %), the UK (51.8 %), and the USA (42%) of the population use TM [66–68]. In those countries, unlike the low- and middle-income countries, the reason for high TM usage is due to the assumption that TMs are safer than allopathic medicines [69].

The most frequently cited TM other than herbal medicine included bone setting, use of the spiritual water (“tsebel”), prayer (faith healing), massage, cauterization, traditional birth attendance, and tooth extraction. This finding is in agreement with reports from other African countries [70].

In our report, most of the TM/herbal medicines were used to treat health conditions experienced during pregnancy, malaria, TB, HIV/AIDS, hypertension, cancer, and the like. Among the users, pregnant women are commonly practicing. As pregnant mothers are more likely to risk groups for potential toxicity derived from herbal remedies which eventually affect the fetus, creating awareness of general use and potential risks of herbal remedies need to be addressed through the health policy system [18].

The current study is highly heterogeneous as observed from *I*^2^. The source of this heterogeneity could be from the methodological quality, geographic and cultural variations, smallness of the included studies, intrinsic variability in the population, and formal synthesis of comparable data. Moreover, eligible studies included in the current study were from some of the administrative regions in Ethiopia and thus may not comprehensively represent the national TM or herbal medicine use. Therefore, to determine the prevalence of TM or herbal medicine at the national level, the large-scale prospective study which represents all administrative regions and city councils should be considered.

In this study, we have collected, compared, and interrogated the dataset of herbal and TM prevalence using systematic reviews and meta-analysis of currently available evidence. Although the studies included may not be from all over Ethiopia, all published TM or herbal medicine prevalence reporting studies from Ethiopia were thoroughly analyzed. Significant heterogeneity observed may be a reflection of the poor methodological quality of included studies and geographical and cultural variations. In addition, all of the included studies were conducted in small particular localities and non-representative convenient sampling techniques were also employed. Therefore, the limitations of the current study arose inherently from the characteristics of the included studies.

## Conclusion

In conclusion, the study revealed that TM/herbal medicine utilization remained an integral source of primary healthcare in Ethiopia. In comparison to the commonly reported prevalence of TM/herbal medicine, there is a considerable decline in TM/herbal medicine prevalence. This might be due to improved access to modern healthcare facilities which could be related to rapid urbanization, slight improvements in rural infrastructures, and public awareness of allopathic medicine. The vast majority who still rely on TM/herbal medicine basically is due to a lack of access to these allopathic medicines with affordable prices. Therefore improving the livelihood of the majority poor and making modern medicines easily accessible with low or affordable prices is highly recommended. The high tendency of TM/herbal medicine use during pregnancy is a finding that is of concern. This calls for urgent regulatory measures from the government and needs to be supported by robust scientific studies for the safety of both the mother and the fetus.

## Data Availability

All data generated or analyzed are included in the manuscript.

## Abbreviations

CAM: Complementary and Alternative Medicine
CI: Confidence Interval
HIV/AIDS: Human Immunodeficiency Virus/Acquired Immunodeficiency Syndrome
TM: Traditional Medicine
SNNPR: Southern Nations, Nationalities and Peoples Region
WHO: World Health Organization
